# Multi-Sequence MRI Registration of Atherosclerotic Carotid Arteries Based on Cross-Scale Siamese Network

**DOI:** 10.3389/fcvm.2021.785523

**Published:** 2021-12-24

**Authors:** Xiaojie Huang, Lizhao Mao, Xiaoyan Wang, Zhongzhao Teng, Minghan Shao, Jiefei Gao, Ming Xia, Zhanpeng Shao

**Affiliations:** ^1^The Second Affiliated Hospital, School of Medicine, Zhejiang University, Hangzhou, China; ^2^School of Computer Science and Technology, Zhejiang University of Technology, Hangzhou, China; ^3^Department of Radiology, University of Cambridge, Cambridge, United Kingdom

**Keywords:** carotid artery, atherosclerosis, multi-sequence MRI, image registration, Siamese network, cross-scale

## Abstract

Cardiovascular disease (CVD) is a common disease with high mortality rate, and carotid atherosclerosis (CAS) is one of the leading causes of cardiovascular disease. Multisequence carotid MRI can not only identify carotid atherosclerotic plaque constituents with high sensitivity and specificity, but also obtain different morphological features, which can effectively help doctors improve the accuracy of diagnosis. However, it is difficult to evaluate the accurate evolution of local changes in carotid atherosclerosis in multi-sequence MRI due to the inconsistent parameters of different sequence images and the geometric space mismatch caused by the motion deviation of tissues and organs. To solve these problems, we propose a cross-scale multi-modal image registration method based on the Siamese U-Net. The network uses sub-networks with image inputs of different sizes to extract various features, and a special padding module is designed to make the network available for training on cross-scale features. In addition, to improve the registration performance, a multi-scale loss function under Gaussian smoothing is applied for optimization. For the experiments, we have collected a multi-sequence MRI image dataset from 11 patients with carotid atherosclerosis for a retrospective study. We evaluate our overall architectures by cross-validation on our carotid dataset. The experimental results show that our method can generate precise and reliable results with cross-scale multi-sequence inputs and the registration accuracy can be greatly improved by using the Gaussian smoothing loss function. The DSC of our Siamese structure can reach 84.1% on the carotid data set with cross-size input. With the use of GDSC loss, the average DSC can be improved by 5.23%, while the average distance between fixed landmarks and moving landmarks can be decreased by 6.46%.Our code is made publicly available at: https://github.com/MingHan98/Cross-scale-Siamese-Unet.

## Introduction

Cardiovascular disease (CVD) is a common disease that causes death worldwide (1). According to the 2019 Global Burden of Diseases and Risk Factors Report released by the World Health Organization, between 1990 and 2019, the prevalence of cardiovascular diseases has steadily increased, almost doubling. In 2019, one third of the global deaths were due to cardiovascular diseases (2). Among them, China has the largest number of deaths from cardiovascular diseases. Atherosclerosis, as the pathological basis of cardiovascular disease, is one of the main causes of CVD, and it is of great significance in the diagnosis and treatment of ischemic vascular diseases (3). The carotid artery is the most common location for atherosclerosis (4). Patients with carotid atherosclerosis (CAS) need to be checked regularly to control the development of the disease.

As the manifestation of atherosclerosis in the carotid artery, CAS is based on the neck atherosclerotic deposits appear on the walls of arteries. Due to the thickening of the walls and lumen, a type of disease characterized by stenosis and decreased blood vessel elasticity occurs (5), the deposits form plaque on the carotid artery. Because the carotid artery is superficial and has less movement, the plaque here is easier to detect. Pathological studies have shown that the occurrence of clinical symptoms of patients is mainly related to the pathological morphology and pathological classification of arterial plaques, among which vulnerable plaques and unstable plaques are the main reasons for the occurrence of clinical symptoms (6, 7). The main pathological features of the reasons include thin fibrous caps, larger lipid cores, and a large number of new blood vessels in the plaques (8). The morphology of vulnerable plaques is mainly a large lipid core under the thin fiber cap, while the morphology of unstable plaques includes plaque rupture, plaque ulcers and calcified nodular plaques (9, 10). Unstable plaque is considered to be the main cause for the occurrence of ischemic stroke (11). Vulnerable plaques and unstable plaques are prone to rupture and hemorrhage under the action of various hemodynamics (12), which leads to internal lipid-rich necrotic core, intraplaque hemorrhage, and surface disruption and in turn leads to the occurrence of clinical symptoms of ischemic stroke (13). Therefore, early analysis and inspection of the number, composition and vulnerability of carotid plaques are essential for the prevention and treatment of ischemic stroke.

Magnetic resonance imaging (MRI) is a leading non-invasive imaging diagnosis and treatment method for carotid atherosclerosis (14). High-resolution MRI can clearly show the external morphological features, internal structural components and location distribution information of plaques. In other words, MRI can identify the carotid plaque constituents *in vivo* with high sensitivity and specificity (15). MRI uses magnetic fields and computer-generated radio waves to create detailed images of internal organs and tissues (16). Cardiovascular-focused MRI can assess structural problems in the aorta (17), such as aneurysms, dissections, vascular inflammation and blockage. Comparing the changes of lesion characteristics at the same location, multi-sequence carotid MRI can obtain different morphological features (18). However, due to the geometric spatial mismatch caused by inconsistency of different sequence parameters and movement deviation of tissues and organs, it is difficult to evaluate the accurate evolution of local changes in carotid atherosclerosis in multi-sequence MRI (19). In addition, during the image acquisition, the position of patient's blood vessel may be different, resulting in the relative bending and distortion of the anatomical structure in the multi-sequence images. Therefore, it is necessary to use medical image registration methods for correction to improve the accuracy of diagnosis.

Medical image registration establishes the correspondence between spatial position and anatomical structure by finding some spatial transformations (20). In recent years, deep learning methods have been applied in medical image registration (21). de Vos et al. proposed a ConvNet network structure (22). ConvNet network uses the Siamese network architecture proposed by Chopra et al. (23). Two Convolutional neural network (CNN) branch networks are used as the input of fixed image and moving image to generate two feature maps. The output parameters of ConvNet network are affine parameters, so the method belongs to affine registration. However, this method only considers the problem of affine deformation, and does not research on deformable registration. For medical images, deformable registration is the main application method of image registration. Hu et al. (24) developed a weakly supervised registration framework for multimodal image registration, which predicts a dense correspondence using labels of anatomical structures. It is suggested that anatomical labels are more reliable and practical. Wang et al. (25) introduced a novel architecture named the constrained affine network (CAN), which combines deformable image registration with affine transformation for multi-sequence MR image registration. The network is also weakly supervised trained with anatomical label to predict a displacement vector field (DVF) between pairs of input images. Although these image registration methods show promising registration accuracy and efficiency, there are still inherent limitations. Due to the different parameters of different image acquisition devices, multimodal medical images often have different image sizes, which leads to crop the image before image registration. It would not only increase the workload of preprocessing, but also since the registration model trained in the same size can only register images of uniforms, it is difficult to apply the trained registration model to practical applications.

The Siamese network was proposed as a deep solution to classification problems, which used discriminative learning methods to extract the key features of training data and to match new samples without prior information (23, 26). The Siamese network is a twin structure of conjoined neural networks. It contains two or more identical sub-networks sharing the convolutional layers, which means that they have the same convolution parameters and weights. So the parameter update during training will be reflected in both of the subnets. In this case, the training parameters of the entire network can be reduced, and the training speed can be accelerated. Therefore, it is appropriate to use Siamese structure for cross-size training. The U-net (27) and its variants (28, 29) was proposed and widely used in medical image processing, as it combines low-resolution and high-resolution information, and has achieved good performance in training with small samples. In this paper, we present a cross-scale Siamese U-net scheme for multi-modal medical image deformable registration. The registration frameworks adopt a weakly supervised learning form, only the anatomical labels are needed for the loss function calculation during the training process, which realizes the effective registration of specific tissue. It takes full consideration of the inconsistent size of deformation fields without introducing additional network parameters. The network uses sub-networks with image inputs of different sizes to extract various features, and a special padding module is designed to make the network available for training on cross-scale features. In addition, to improve the registration performance, a multi-scale loss function under Gaussian smoothing is applied for optimization.

## Materials and Methods

### Datasets and Pre-processing

We use the clinical carotid artery data set collected from our collaborative hospital to conduct experiments to evaluate the proposed registration method. This clinical carotid artery data set contains 11 cases of carotid atherosclerosis three-dimensional carotid MRI images, and each patient has several different sequences to analyze the development of atherosclerosis. Patients attending the stroke unit and neurovascular clinics in hospital with carotid artery disease were invited to participate in the study. Only those patients who can give written informed consent were recruited. Patient demographic and clinical measurements were also recorded. The criteria for inclusion are: (1) internal carotid artery stenosis of 30–69% on duplex imaging during screening assessment; (2) normal sinus rhythm, confirmed by 24 h Holter monitoring and normal transthoracic echocardiography. Exclusion criteria include: (1) previous CEA of the index carotid artery; (2) cardiac arrhythmias; (3) a known coagulation/clotting disorder potentially responsible for the patient's symptoms; (4) patients having received thrombolysis for the ictal event; and (5) clinical contraindications to MRI such as inner ear implants, metallic implants and cardiac pacemakers.

A fast spin echo (FSE, CUBE) was performed on a 1.5T MR system and MR images were captured using a bilateral four-channel phase aligned carotid surface coil (PACC, Machnet BV, Elde, The Netherlands). This batch of images contains three main modes: T1-weighted sequence (T1), CUBE sequence (T1GD) using Gd contrast agent, and three dimensional TOF sequence. The imaging parameters of T1 and T1Gd CUBE sequences were as follows: Field of view: 14 × 14 cm^2^, TR/TE: 400 ms/10.8 ms, slice size: 512 × 512, slice resolution: 0.2734 × 0.2734 cm^2^, slice thickness: inserted to 0.6 mm, including the number of slices in coronal position: 64–72, image space coordinate system orientation :RSA (Right, Anterior, Superior). The parameters of TOF are as follows: Field of view: 14 × 14 cm^2^, TR/TE: 29.2 ms/3.3 ms, slice size: 256 × 256, slice resolution: 0.5469 × 0.5469 cm^2^, slice thickness: inserted to 1 mm, including the number of slices in the coronal position: 56. Orientation of image space coordinate system: RAI (Right, Anterior, Inferior). We select T1GD and TOF sequences for image registration experiments. T1GD sequence is a fixed image, and TOF sequence is a floating image.

**Figure 1:**
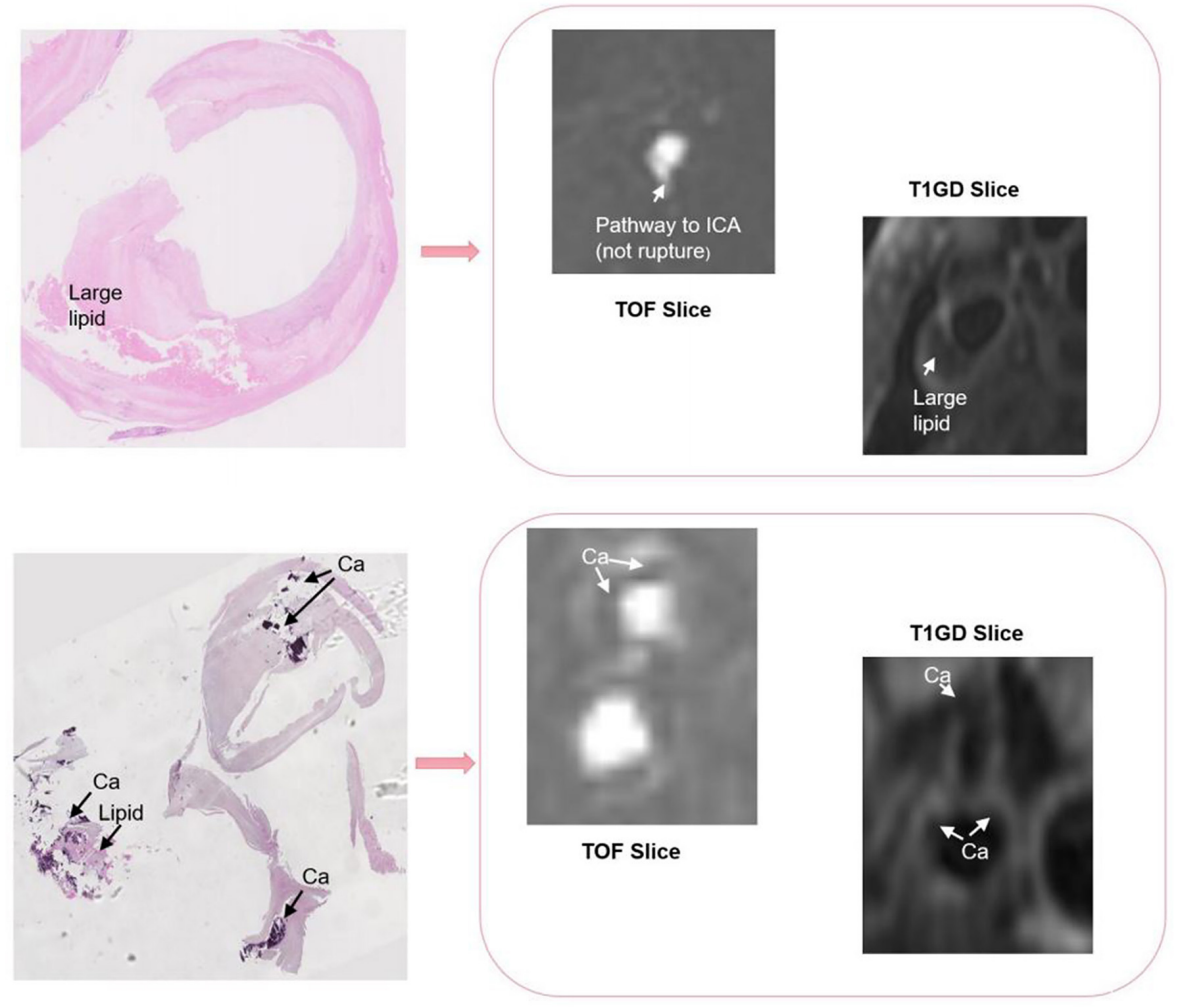
The lesion in a carotid artery.

**Figure 2:**
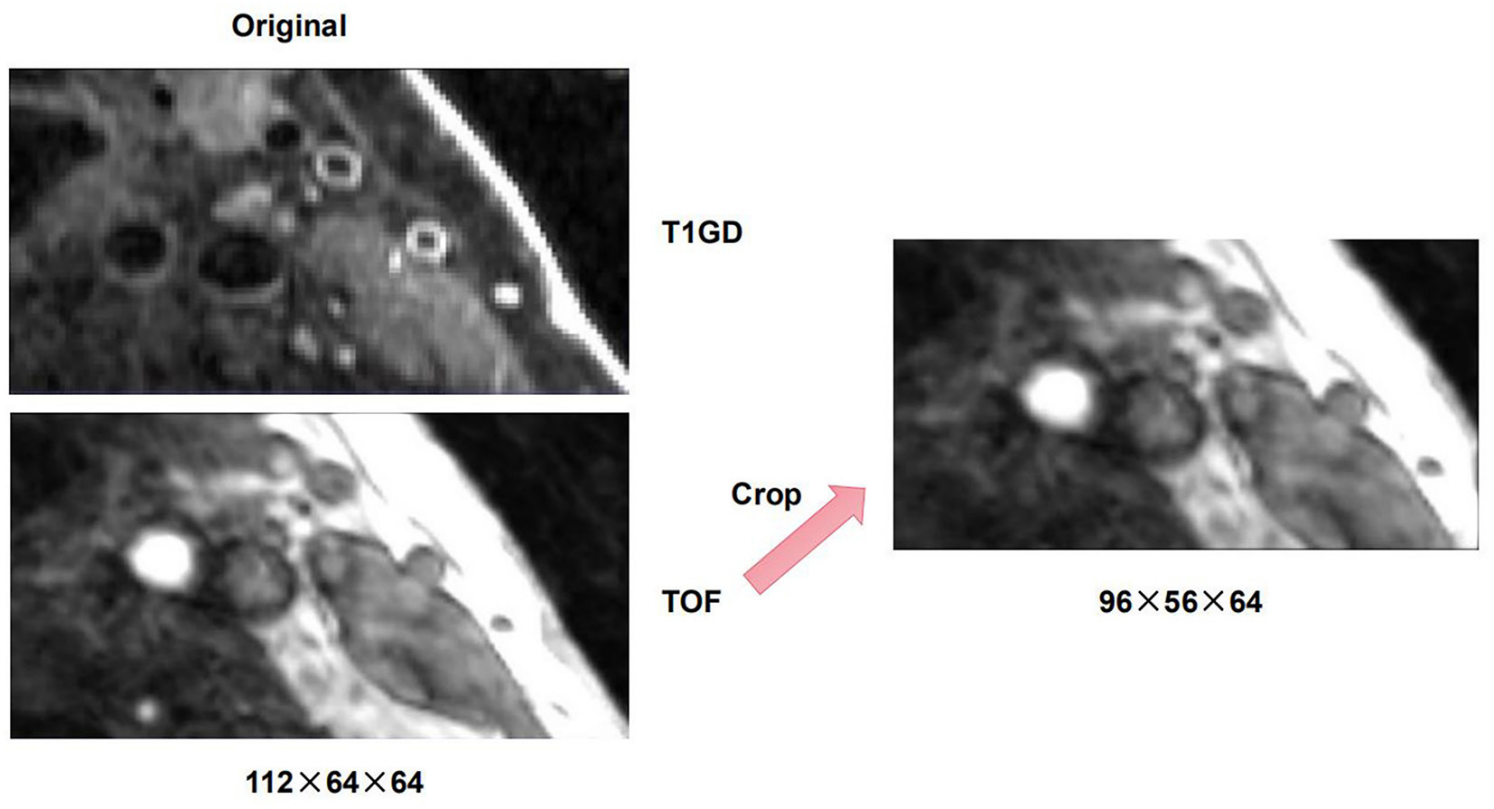
An example of cropped carotid artery MR sequences.

There are differences in resolution between different modalities of multi-modal arterial data, so it is necessary to perform preprocessing operations on the original data. To remove the intensity unevenness between different images, we apply N4 bias field correction to correct the selected sequence of images (30). After that, linear resampling is used to interpolate all sequence images, and the voxels of all sequence images are unified to the size of 0.2734 × 0.2734 × 0.2734. According to the carotid artery position of each patient, all carotid artery images are adjusted and cropped to a uniform size by removing the outer boundary of the image. The cropping size of different patients is the same. After that, we cut the three-dimensional carotid artery MRI images of 11 patients with carotid atherosclerosis into two halves, and deleted two abnormal right carotid artery data. Then, linear resampling is again performed to 0.6 mm voxel spacing. Finally, we obtained a total of 20 pairs of MR images. On this basis, in order to verify the purpose of the research, we obtain a suitable cropping area for each patient's data of the carotid artery according to the position of the carotid artery, so as to prevent the carotid artery part in the image from being cut off and causing the distortion of the images. The original image size of T1GD and TOF sequence is 112 × 64 × 64, we adjust the size of TOF sequence to 96 × 56 × 64 by cropping in cross-size experiments. An example of the carotid artery lesion is shown in Figures 1, 2 shows an example of cropped carotid artery MR sequences. The upper part corresponds to the original multi-modal registration image, and the lower two images only crop a single floating image TOF sequence.

**Figure 3:**
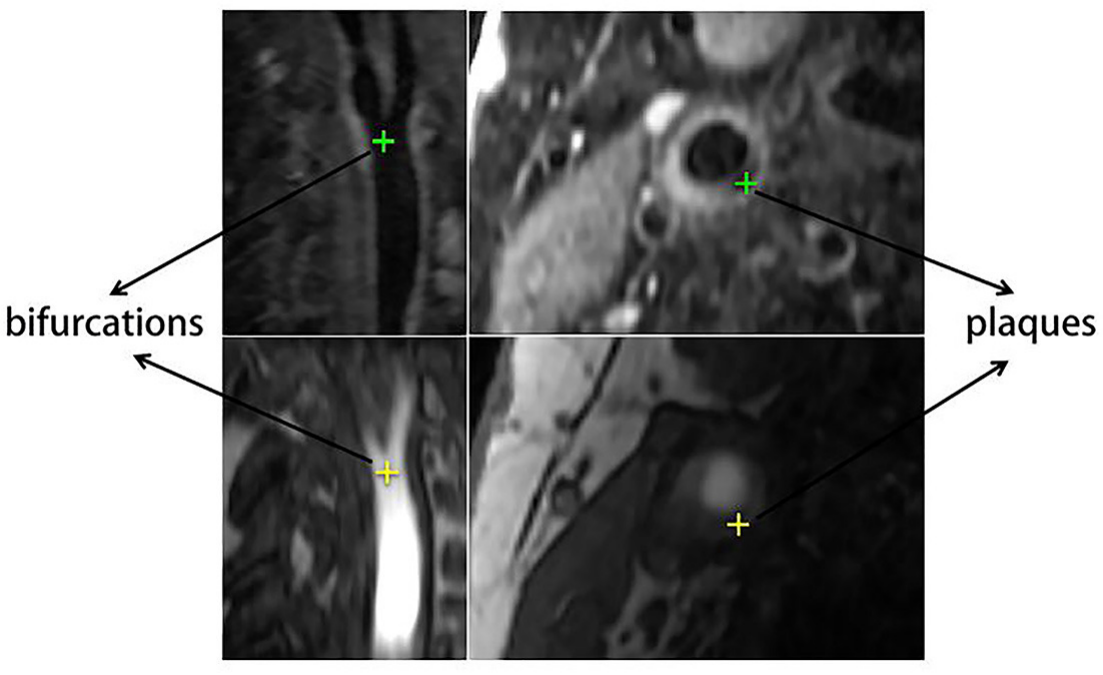
Example of labeled carotid bifurcations and plaques.

From the patients, 20 pairs of corresponding anatomical landmarks were labeled and registered by two doctors and two research students using ITK-SNAP (31) on the original image data, and all were confirmed by two second observers including a radiologist and a research expert. The lumens of carotid arteries on original T1GD and TOF slices were segmented automatically first using cascaded residual U-Net (32) and then edited and confirmed manually. Besides full lumen segmentations for all cases, the landmarks also include the artery bifurcations and lesions as shown in Figure 3. The landmark annotation process took more than a month. Among all the anatomical labels, we used the lumen labels for both training and validation. For each patient, only one position was labeled as the bifurcation, and several position points on different slices were selected for the plaque according to the location and size of lesion. The bifurcation and plaque labels were only used for evaluation.

Additionally, to verify the generalizability of our algorithm, we also evaluate the registration model on brain MRI scans from the Brain tumor Segmentation (BraTS) 2020 challenge (33). The training set contains 373 cases of brain MRIs with the patients' tumor segmented manually. There are four contrasts (T1Gd, T1c, T2, T2-FLAIR) used in the study, and the size of images is 240 × 240 × 155 with 1 mm isotropic resolution in a standardized axial orientation. We choose T1c and T2 to form 80 pairs of fixed images and moving images for experiments, 60 pairs for training and 20 pairs for testing. All raw scans are down-sampled to the size of 96 × 96 × 64. Specially, we remove the edema portion of the tumor segmentation to focus on the tumor structure. As the raw scans in BraTS are well-aligned, we transform the T2 images with random synthetic deformation fields generated by using elastic deformations followed by Gaussian smoothing (34). In this way we produce reasonable mis-alignments and the synthetic deformations are used as ground truth for evaluation.

### Registration Based on the Siamese U-Net

Image registration refers to finding a spatial one-to-one mapping relationship from voxels in one image to voxels in another image. The former image that needs to find the spatial transformation mapping relationship is denoted as the moving image *I*_*M*_*(x)*, and the latter that does not need to find the transformed image is called the fixed image *I*_*F*_*(x)*. The dimensions of the fixed image *I*_*F*_*(x)* and the moving image *I*_*M*_*(x)* are set to *d*, and they are respectively defined in the spatial domain of their respective images and set as: Ω_*F*_ ⊂ *R*^*d*^ *and* Ω_*M*_ ⊂ *R*^*d*^. The registration network looks for a displacement function *u(x)* to obtain the spatial mapping relationship, so that it is aligned with *I*_*F*_*(x)* and *I*_*M*_(*x* + *u*(*x*)) in the space domain. The above registration is considered to be a process of finding a conversion mapping *T(x)* = *x* + *u(x)*, which is a mapping from a fixed image to a moving image *T(x)*: Ω_*F*_ ⊂ *R*^*d*^ → Ω_*M*_ ⊂ *R*^*d*^. In order to ensure the alignment accuracy of the registration, a penalty constraint term needs to be added as the cost function *L*, Therefore, the registration problem can be expressed as an optimization problem. The goal is to minimize the cost function *L*. The formula can be expressed as:


(1) $$\begin{array}{r} \tilde{T}=\arg\underset{T}{\min}L\left(I_{F},I_{M},T\right). \end{array}$$

The cost function is called the loss function. According to different data types, different metrics are selected as the loss function. The optimal registration effect can be obtained through continuous iterative optimization. Therefore, the essence of image registration is an iterative optimization problem of finding the optimal solution.

In this paper, Siamese network is used to construct a registration network that can realize image registration between different sizes. Different from the general convolutional neural network, the input of the Siamese network includes two image channels, and the output is the similarity between the two images. When the Siamese network is used for image registration, the dual channel of the Siamese network is used to extract the bottom-level feature information of the input image, and then the bottom-level feature information is normalized. In particular, the models used for feature extraction of two images share weights to ensure that the deep features of the two images can be obtained under the same metric. The U-net network has proven to show good performance in many medical image tasks due to unique structure (27, 35, 36), so the registration structure designed is modified on U-net model. The down-sampling structure is similar to Siamese network (37). However, the up-sampling structure is added to the network that form a model similar to the U-Net structure.

**Figure 4:**
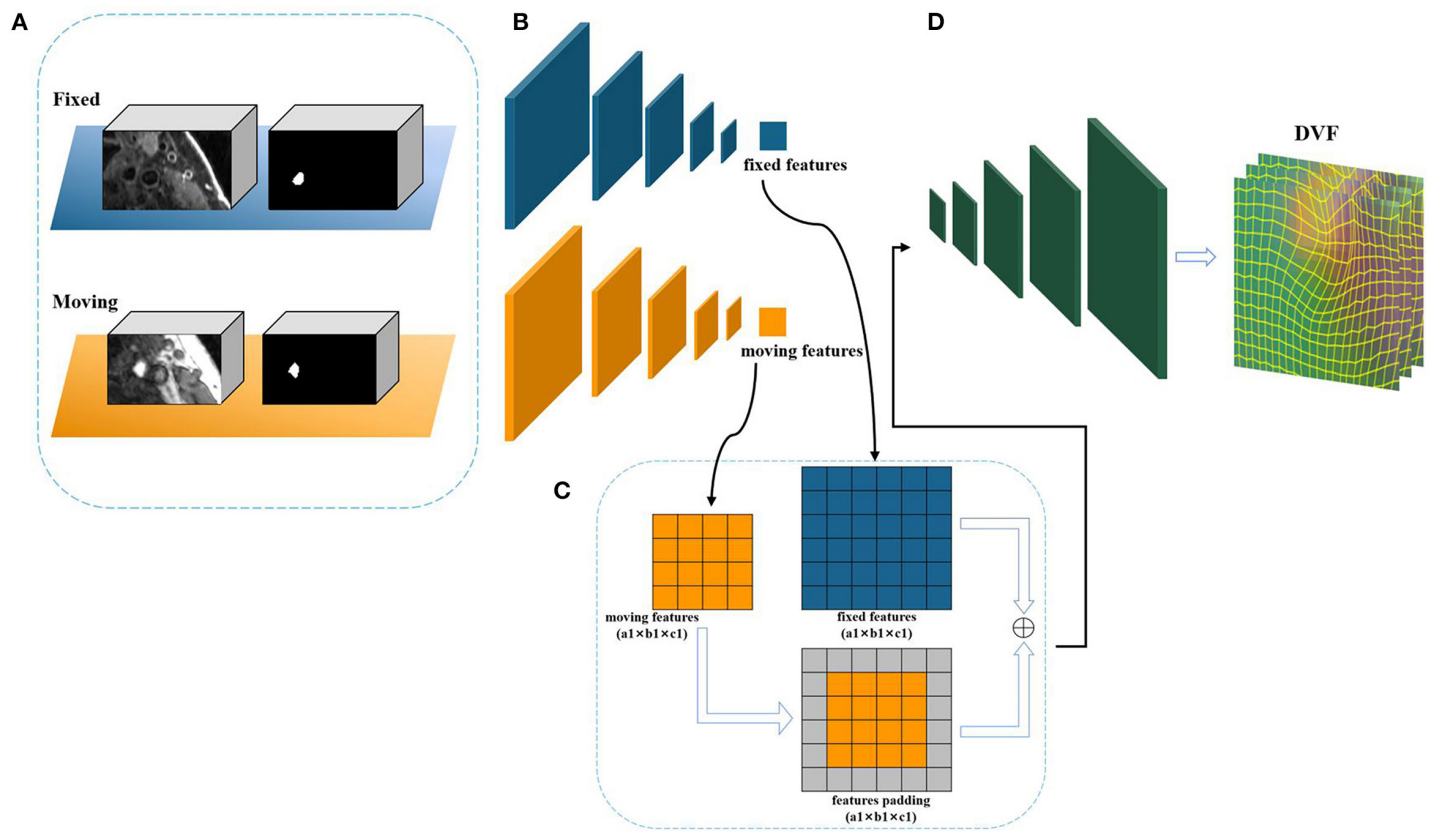
The framework of cross-scale Siamese U-Net. **(A)** The inputs of Siamese U-Net containing an image pair and a label pair. **(B)** The feature extraction sub-network of down sampling. **(C)** The padding module. **(D)** The up-sampling structure to restore the feature size of deformable displacement field.

As mentioned above, the Siamese-based network framework is transformed from the U-Net structure, and uses the same structural parameters as the U-Net network to learn the displacement vector field between the fixed image and the moving image. Figure 4 shows the internal details of the network structure of cross-scale Siamese U-Net, the network has two down-sampling encoders and one up-sampling decoding structure frame and skip connection. The down-sampling encoder can obtain the multi-scale features of each different level of the input image, and can obtain the image context information. The information is important for the transformation of registration, which make the network can obtain the non-linear transformation information from the moving image to the fixed image. In the figure, a 7 × 7 × 7 kernel used in the first convolution layer to expand the receptive field. In addition, each down-sampling convolution module has two concatenated 3 × 3 × 3 convolutions and a 2 × 2 × 2 max-pooling as the down-sampling space operation. Unlike common registration methods based on convolutional neural networks, the input is added to the anatomical label corresponding to the registered image as auxiliary information to obtain the location of organs to be registered. The sub-network uses the weight sharing structure of the Siamese network to make the fixed image network and the moving image network obtain the same network parameters. At the same time, the size of the registered image is restored by sampling up the decoding path, and the restored size is the same as the fixed image, so that the correspondence between each voxel can be established from the pair of registered images. In addition, the skip connection still needs to obtain the same feature size as the up-sampling through the filling module, which is used to make up and restore the image information, and the padding method of the skip connection is unified as edge zero-value padding. Finally, a convolution operation is used for dimensionality reduction and feature fusion, and the three-dimensional displacement vector field between the registered image pairs is obtained.

### Cross-Size Registration

In the previous section, the registration framework of the Siamese U-Net structure was introduced. In this section, we will introduce the basic principles of using this framework to achieve registration of cross-size inputs.

During the convolution operation of the convolutional neural network, the output shape of the convolutional layer is determined by the shape of the input and the convolution kernel. In a certain convolutional layer, suppose the input feature shape size is *n*_*h*_ × *n*_*w*_. The size of the convolution kernel is *k*_*h*_ × *k*_*w*_, then we will get the output feature shape size is *(n*_*h*_ *- k*_*h*_ + *1)* × *(n*_*w*_ *- k*_*w*_ + *1)*. So, if we apply many consecutive convolutions, we will get an output much smaller than the input, and eliminate any interesting information on the original image boundary. Using padding to deal with this problem is the most effective way. Padding is a term related to convolutional neural networks, which refers to the number of pixels added to the image when the CNN convolution kernel processes the input. For example, if the padding in the CNN is set to zero, then the value of each pixel added will be zero, However, if the zero padding is set to 1, a pixel boundary will be added to the image, where the pixel value is 1, and the convolution operation of the convolutional neural network usually needs to be filled by default. Since the branch network of Siamese U-Net has the same structural parameters, so the input images are different, the two outputs will inevitably get different feature sizes. Using the padding method of the convolutional neural network to fill the two features make the feature size consistent.

There are many existing padding methods, including zero padding, boundary copying, mirroring and block copying. The registration method of the weakly supervised learning registration is mainly aimed at a certain tissue of the image for effective registration, and the anatomical parts that need to be registered rarely have image boundaries (24). So, the corners and borders of these images rarely play a role in the registration process. At the same time, considering the need to maintain the original feature information as much as possible to reduce the impact of feature errors caused by padding, only the padding method of zero padding is used to supplement the boundary. The specific details of this method in the Siamese U-Net structure. The structure use two padding methods, namely symmetrical padding and single-edge padding, the feature padding method is selected according to the size and position corresponding to the input registration images. If the image size is uniformly different on the edge, then the symmetrical method is used. If the image size differs on a single edge, use single edge padding.

It is worth noting that the proposed padding module is placed between the down-sampling and up-sampling structure and is not placed before the up-sampling network input for padding. Regarding the conditions of the stitching operation, placing the padding module before the up-sampling can also achieve image registration of different sizes. However, it will have an adverse effect on the accuracy and timeliness of the network model. Then, the reasons are as follows. First, the registration network chooses a twin network based on the 3D U-Net (38). This structure is still a typical encoding-decoding structure. The encoder obtains the underlying semantic features through multiple down-sampling. These underlying semantic features are connected through skip connection (36), so that the feature maps recovered from the up-sampling merge with more underlying features. However, if the original input image is pad, it will affect the underlying features of the original image, and reduce the accuracy of the registration model. Second, the registration model is based on an end-to-end image registration framework, if the network training is carried out in this way, the predicted displacement vector field has to be pad with each registered image pair in order to meet the model parameters of the sampling structure on the training network, generate the corresponding displacement vector field. The method is almost a disguised preprocessing operation on the original image, which violates the principle of ensuring the registration on the basis of the original image, and affects the timeliness of the registration.

### Gaussian Smoothing Optimization

The registration structure based on Siamese network needs to use dice as the loss function to calculate the similarity between fixed image and moving image labels. The loss function of Dice coefficients is as follows:


(2) $$\begin{array}{r} L_{DSC}\left(p,g\right)=1-\frac{2pg}{p+g}, \end{array}$$

where *p* represents the binary label corresponding to the predicted segmentation pixel, *g* represents the binary label corresponding to the ground truth pixel. This formula is equivalent to the ratio of the intersection and union of the segmented area node predicted by the network and the ground truth. It uses the pixels of the same category in the foreground area as a set relationship to calculate the loss function. This calculation method ignores a large number of background pixels and solves the problem of unbalanced training data, so the convergence speed is faster, and achieve an ideal performance in the segmentation task. However, there may be some problems for the registration task. The gradient form obtained by deriving *L* is as follows:


(3) $$\begin{array}{r} \frac{\partial L_{DSC}\left(p,g\right)}{\partial p}=\frac{2g^{2}}{{\left(p+g\right)}^{2}}. \end{array}$$

**Figure 5:**
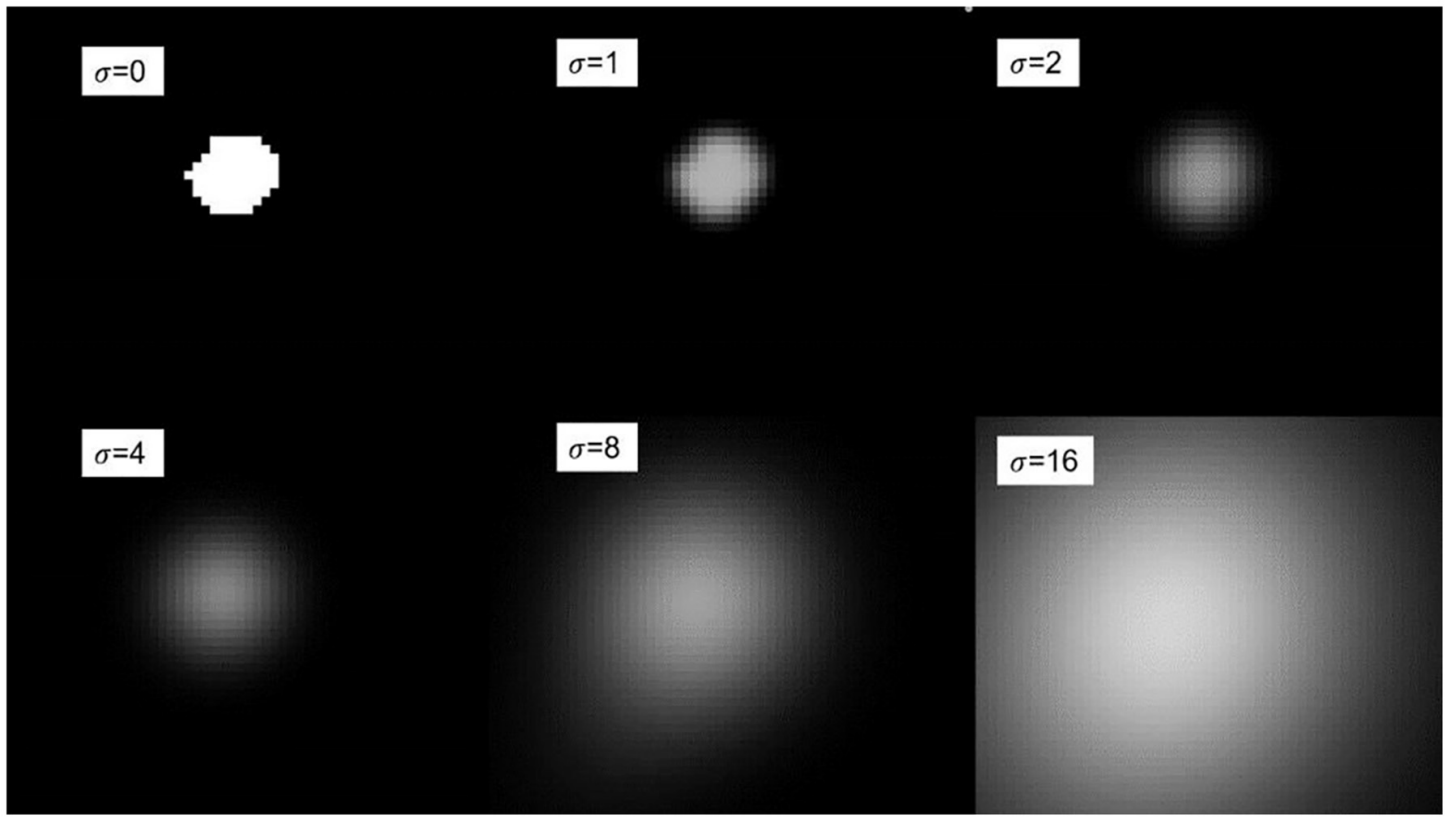
Schematic diagram of Gaussian blur under different variances.

It can be seen from the gradient formula that there is a square term in the denominator. However, in medical image registration, there are often mismatches or incomplete matches between the anatomical labels of the registered images. At the same time, there are few anatomical labels of the structure to be registered, which will cause these values to be small. In this case, the obtained gradient will be very large, resulting in the unstable training, so the registration network will be difficult to obtain adaptive parameters. In order to overcome this problem and obtain an ideal registration model, the Gaussian smoothing (39) method is used to make the anatomical labels of the registered image have a reasonable smooth blur, and the multi-scale dice method is used to optimize the loss function. The Gaussian fuzzy kernels in the convolutional neural network is used to perform Gaussian smoothing on the anatomical labels, which is defined as follows:


(4) $$\begin{array}{r} k\left(x\right)={\frac{1}{2\pi \sigma^{2}}e}^{\frac{-x^{2}}{2\sigma^{2}}},\;x\in \left[-3\sigma ,\;3\sigma +1\right), \end{array}$$

where σ refers to the standard deviation. According to the Gaussian function, given different variances σ, different fuzzy kernels will be generated. Using the range of the variance value σ, we can obtain 6σ positive and negative symmetrical values, and then divide each obtained value by the sum of all values to obtain the final required fuzzy kernel, Which is used as the convolution kernel parameter of convolution, so that the generated blur kernel can perform convolution operation on the anatomical label. Figure 5 shows the Gaussian blur display under different variance values σ. It can be seen in the figure that when σ is 0, no blur kernel is generated, and the fuzzy convolution operation cannot be performed. As the variance value σ gradually increases, the degree of blurring becomes higher. The loss function of multi-scale dice under Gaussian smoothing is as follows:


(5) $$\begin{array}{r} L_{GDSC}\left(p,g\right)=1-\frac{2p_{z}g_{z}}{Z\left(p_{z}+g_{z}\right)},\;z\in \sigma \;, \end{array}$$

where *Z* is the number of scales, and *p*_*z*_ and *g*_*z*_ respectively represent the moving and fixed binary segmentation labels of the registered image after Gaussian smoothing. We use an isotropic Gaussian filter to effectively extract the spatial information of the binary segmentation label, the σ = {0,1,2,4,8} set size used in the experiments. For different variance values σ, a larger Gaussian kernel will promote the global convergence of the entire deformation field training network, while a smaller Gaussian kernel will reflect the details of the deformation field.

In general, using various Gaussian filters to perform Gaussian smoothing to calculate the loss function of the anatomical label is very important for the accuracy of the medical image registration model. The smoothed anatomical label balances the gradient between the foreground and the background and provides a non-saturated gradient to prevent the gradient instability in the training process.

## Experiments and Results

### Data Augmentation

CNN has shown high-performance in medical image registration. However, these networks rely heavily on a large amount of data to train the network model parameters in order to correctly weigh the functions of each layer, because medical images cannot be artificially produced compared to natural images, they can only be derived from clinical patients. At the same time, the image labels require a very high level of medical expertise, so it is difficult to obtain accurately labeled medical image data. In this case, data augmentation technology to expand the training data set has more important practical significance for medical images.

In this paper, there are two main reasons for the need of data enhancement in carotid image registration. First, it is difficult to obtain a registered image with strictly consistent image information in clinical practice, and different scanner types may cause the obtained data to have small directional differences. Second, the data collected clinically are mainly individual cases, so the data obtained has a high degree of freedom, and insufficient data representation will lead to insufficient fitting in the experiment. In order to solve the problem that the number of data sets affects the generalization ability of the model, and to improve the accuracy and robustness of the model, we mainly adopt two data enhancement methods. One is to use the left and right flip method to increase the number of training data before network training, and the other is to use affine enhancement technology during network training.

### Loss Function

The displacement vector field output by the registration framework based on the Siamese network not only needs to ensure the accuracy of the registration part, but also ensure the smoothness of the entire displacement field, so as to ensure the medical rationality of the overall registered image. So the designed loss function consists of two parts, one of them is dice y multi-scale Gaussian smoothing as a measure of label similarity, and the other is a penalty constraint term for smoothness. The formula is as follows:


(6) $$\begin{array}{r} L=L_{GDSC}+\alpha L_{smooth}, \end{array}$$

where *L*_*smooth*_ is the smoothness regular term of the displacement vector field, which is used to constrain the displacement vector field in training so that the network can obtain a reasonable output. *L*_*smooth*_ is defined as follows:


(7) $$\begin{array}{r} L_{smooth}=\sum_{P\in \Omega }||\nabla \varphi \left(p\right)||, \end{array}$$

where Ω represents the space domain of the displacement vector field, φ is the displacement vector corresponding to each voxel P in Ω. So, the regular term is essentially a gradient operation at each point of the vector field.*L*_*smooth*_ use the bending energy as the constraint item, and α is the regularization super-parameter. In the experiment, the parameter α is set to 0.5.

### Experiments and Results

#### Experimental Settings and Evaluation

In our experiments, considering the limitation of insufficient image data, we performed 4-fold cross-validation to evaluate the performance of the model. After the preprocessing and data augmentation, we obtained 20 pairs of carotid MR sequences from 11 patients with carotid atherosclerosis, including 11 pairs of left carotid arteries and nine pairs of right carotid arteries. If both the right and left carotid artery data from a given patient are used, we must ensure that both sides were in the same training or test sets to prevent data leakage between the training set and test set. We performed 4-fold cross validation both in ablation experiments and contrast experiments. Our method is trained using TensorFlow (40) with a single Nvidia Geforce GTX 1070Ti. The Adam Optimizeris (41) applied for training with an initial learning rate of 1e-4. Typically, we set the number of iterations to 10,000 and save the model every 15 iterations, where the batch size is set to 2.

In order to evaluate the effectiveness of the proposed Siamese registration framework, various evaluation indexes are set. For the quantitative analysis of experimental results, it is impossible to clearly evaluate the transformation parameters of the obtained displacement vector field. Therefore, there is no unified evaluation for image registration. We use the Dice similarity coefficient (DSC) (42, 43) to quantify the accuracy of the registration algorithm, and the registration time to quantify the timeliness of the registration algorithm. In addition, for the multi-modal carotid artery data, landmarks are used as the evaluation criteria for key points of tissue locations. Landmarks include carotid artery bifurcations and plaque locations. We calculated the average distance between the fixed landmark and the moving landmark (Lm.Dist). At the same time, the registration time is tested on the GPU as one of the metrics to measure the registration performance. For the evaluation of BraTS data, in addition to DSC, we also evaluate the accuracy of registration by calculating the Target Registration Error (TRE) (44), that is the difference between the registration displacement vector field obtained by the network output and the ground truth.

## Results

In order to accurately evaluate the designed registration network performance. In the experiment, we applied U-Net, Attention U-Net (28) and MultiRes U-Net (29) networks as basic structure for training. The network is designed as a branch structure, called Siam U-Net, Siam Attention U-Net and Siam MultiRes U-net, the networks have the same convolutional structure parameters, and the padding module integrate into the networks. We compare these novel structures with their original formal, and we also compare against these networks together with the constrained affine network (CAN) (25), which combines deformable image registration with affine transformation for multi-sequence MR image registration.

Table 1 shows the evaluation values of different Siam U-Net network models after registration on the carotid dataset in the case of both equal-size input and cross-size input. The results show that the Siamese structures with our padding module can achieve roughly the same registration effect whether the data is trimmed or not. For Siam U-Net and Siam Attention U-Net, the DSC drops a little when the TOF images are cropped into different size. But for Siam MultiRes U-net, the DSC goes a little up after the cropping. There is also no significant change in the computation time with cropped input. The average variations are about 0.08, 7.58 and 3.08% in DSC, Lm.dist and computation time respectively. Among these networks, Siam AttentionUnet achieves the best result in the accuracy of registration with cross-size input, with an average DSC of 0.841, while Siam U-net achieves the best result in Lm.dist and time, which are 1.127 and 0.239 respectively.

**Table 1:** Registration performance of cross-size carotid artery input.

**Network**	**Siam U-Net**		**Siam Attention U-Net**		**Siam MultiRes U-Net**	
	**Original**	**Cropped**	**Original**	**Cropped**	**Original**	**Cropped**
DSC (%)	0.825	0.816	**0.841**	**0.833**	0.741	0.756
Lm.Dist (mm)	**1.127**	1.349	1.305	**1.273**	1.339	1.435
Time (s)	**0.239**	**0.275**	0.297	0.311	0.405	0.384

*All the best results are in bold*.

To verify the performance and generalizability of the algorithm, we evaluate the Siamese based U-Net models with CAN based U-Net models on both the carotid artery dataset and the BraTS dataset. The experimental results are shown in Table 2. It shows that our proposed Siamese based U-Net models can achieve equal results with CAN based U-Net models in both registration tasks. Specially, Siam Attention U-net can also obtains the best DSC on the BraTS dataset.

**Table 2:** Registration performance of different networks on carotid artery and BraTS datasets.

**Network**	**Carotid artery**			**BraTS**		
	**DSC (%)**	**Lm.Dist (mm)**	**Time (s)**	**DSC (%)**	**TRE (mm)**	**Time (s)**
Unet	0.767	0.954	0.267	0.848	2.045	0.627
MultiResUnet	0.762	1.418	0.284	0.794	4.856	0.581
AttentionUnet	0.823	1.262	0.232	0.858	1.966	0.632
Unet + CAN	0.833	0.757	0.241	**0.869**	1.812	0.612
MultiResUnet + CAN	0.811	1.421	0.217	0.821	**1.070**	0.679
AttentionUnet + CAN	0.839	**0.692**	**0.184**	0.862	1.886	**0.570**
Siam Unet	0.825	1.127	0.239	0.827	2.243	0.781
Siam MultiResUnet	0.741	1.339	0.405	0.829	2.906	0.773
Siam AttentionUnet	**0.841**	1.305	0.297	0.867	2.095	0.703

*All the best results are in bold*.

**Figure 6:**
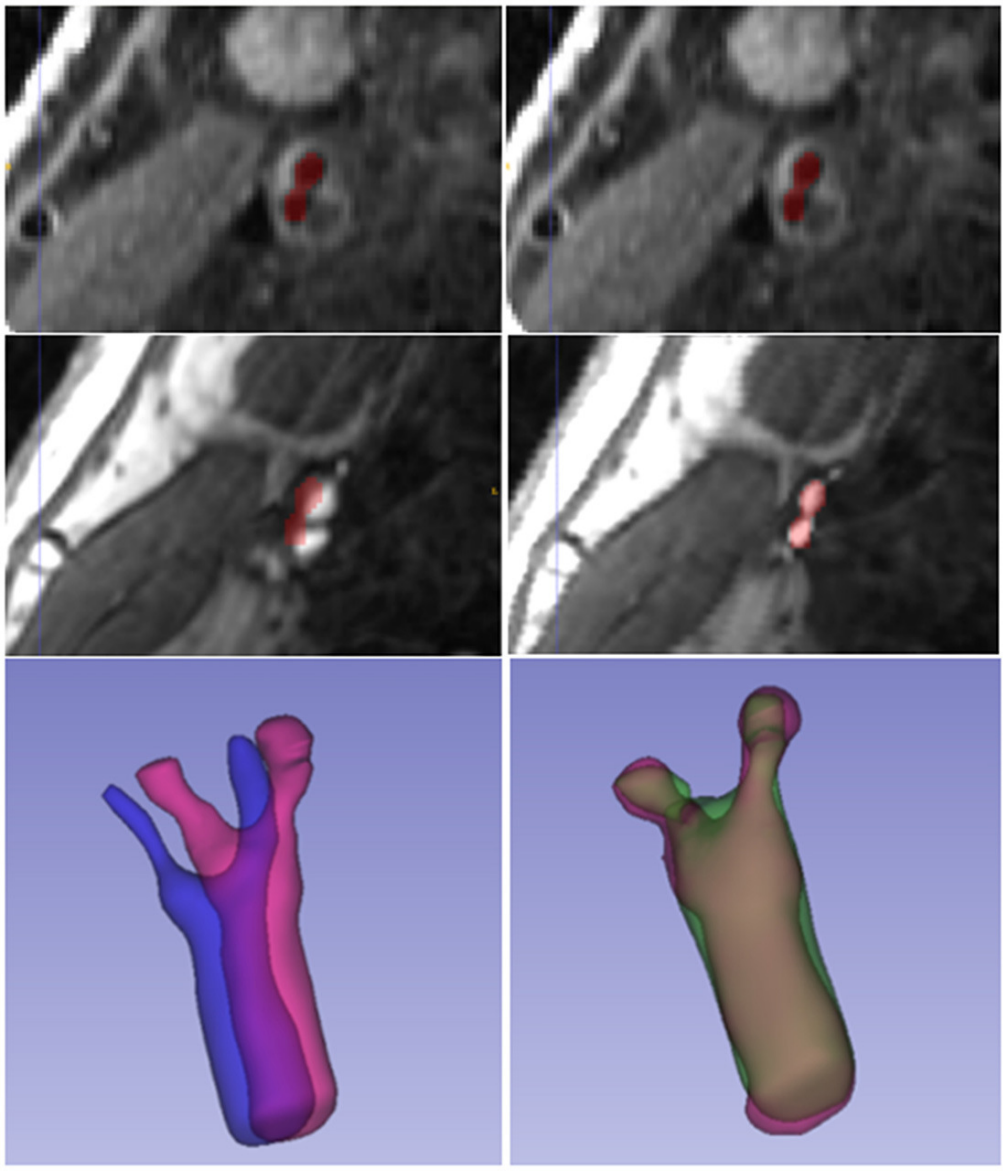
3D visualization of atherosclerotic carotid artery before and after registration.

**Figure 7:**
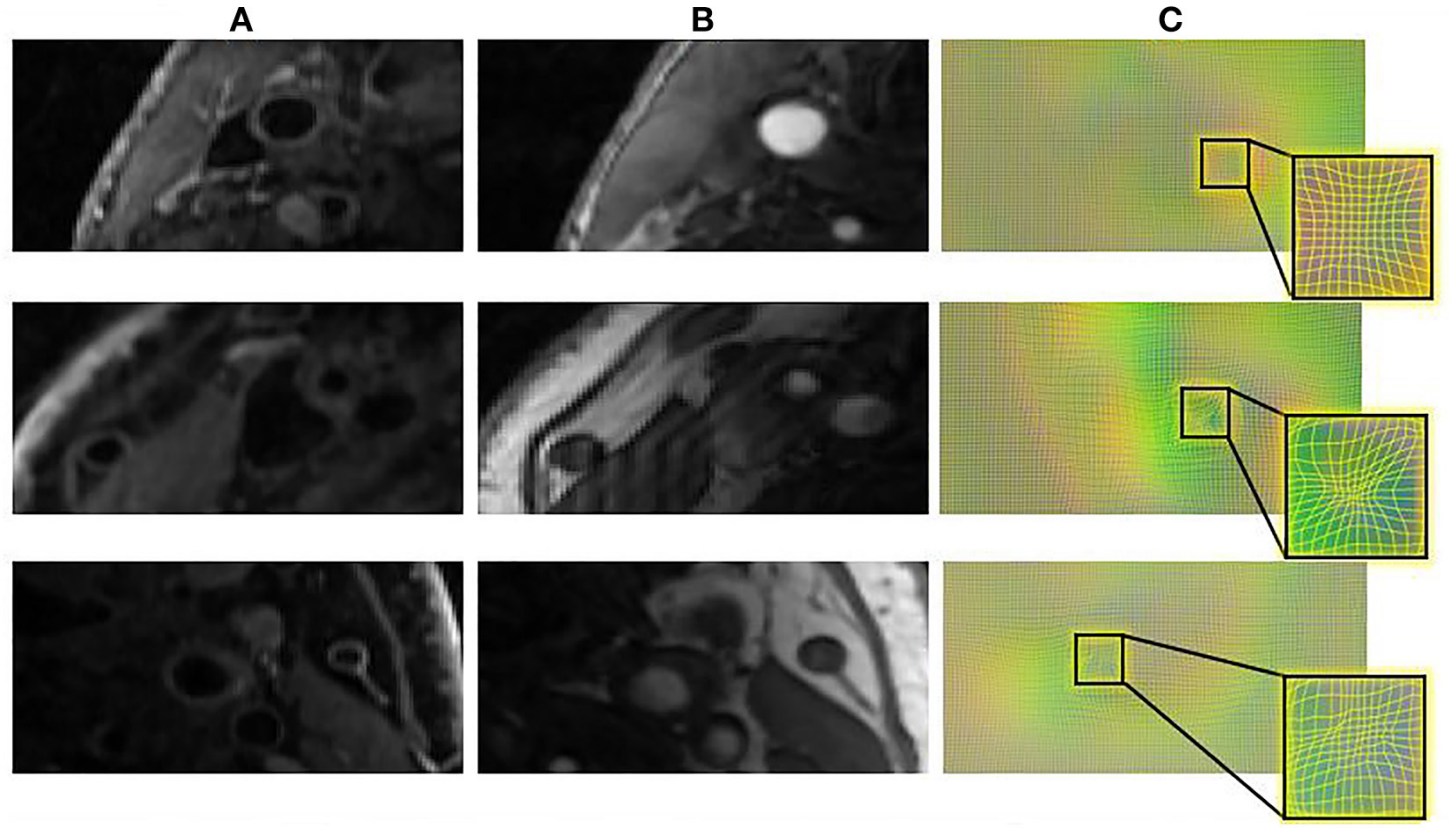
The DVF visualization of carotid artery slices before and after registration. **(A)** The input T1GD image **(B)** the input TOF image **(C)** DVF visualization after registration.

Figure 6 displays a 3D visualization of atherosclerotic carotid artery before and after registration. This data uses the bifurcation position with a DSC value of 0.889 after registration. The label used in the figure is the carotid artery vessel label of the fixed image T1GD sequence. Figure 7 shows the visualization of deformation vector fields (DVF) of some examples after registration.

In the experiment of verifying Gaussian smoothing optimization (GDSC), we compare the registration performance in U-Net, Attention U-Net, and MultiRes U-Net networks and compare them with dice loss function during the training of each registration network to analyze the optimized loss function performance. We also compare it with the dt loss proposed in Wang et al. (25), which describes the center point distance of the anatomical labels. The experimental results of the registration are shown in Table 3. As shown in the table, the performance of GDSC on the three U-Net based networks is relatively better, especially there is an obvious improvement on the MultiRes U-net network. Compared with original DSC and dt loss, by using GDSC, the DSC is increased by 5.4 and 1.0%, respectively, while the Lm.dist is decreased by 14.3 and 23.0%, respectively. The results show that compared with the DSC loss function, the GDSC loss function can provide more effective model optimization capabilities in the three networks, and the registration performance has been improved.

**Table 3:** Registration performance of carotid artery under different loss functions.

**Network**	**DSC (%)**			**Lm.Dist (mm)**			**Times (s)**		
	**+ DSC**	**+ dt**	**+ GDSC**	**+ DSC**	**+ dt**	**+ GDSC**	**+ DSC**	**+ dt**	**+ GDSC**
Unet	0.767	**0.837**	0.825	0.954	**0.900**	1.103	0.267	0.213	**0.184**
MultiResUnet	0.762	0.795	**0.803**	1.418	1.578	**1.215**	**0.284**	0.297	0.307
AttentionUnet	0.823	0.843	**0.847**	1.262	**0.704**	1.081	0.232	**0.187**	0.249

*All the best results are in bold*.

Additionally, we also conduct an ablation study using different Gaussian fuzzy kernels by adjusting the variance set. As shown in Table 4, the variance set {0,1,2,4,8} we selected achieves relatively good results on the three networks. Taking the AttentionUnet as an example, compared with using the other three variance sets, the DSC is increased by 1.9, 2.3 and 0.6%, while the LM. Dist is decreased by 10.3, 11.18 and 1.8%, respectively.

**Table 4:** Registration performance of carotid artery using different variance set.

**Network**		**Unet**	**MultiResUnet**	**AttentionUnet**
σ = 0,1	DSC (%)	0.785	0.766	0.831
	Lm.Dist (mm)	1.180	1.437	1.205
	Time (s)	0.241	**0.291**	0.257
σ = 0,1,2	DSC (%)	0.803	0.783	0.828
	TRE (mm)	1.206	1.393	1.217
	Time (s)	0.236	0.315	0.251
σ = 0,1,2,4	DSC (%)	**0.831**	**0.807**	0.842
	Lm.Dist (mm)	**1.026**	1.223	1.101
	Time (s)	0.217	0.302	**0.244**
σ = 0,1,2,4,8	DSC (%)	0.825	0.803	**0.847**
	Lm.Dist (mm)	1.103	**1.215**	**1.081**
	Time (s)	**0.184**	0.307	0.249

*All the best results are in bold*.

## Discussion

MR medical images have the advantages of high anatomical tissue resolution and repeatability. Carotid MRI is an important image morphology for identifying carotid artery vascular morphology and quantifying carotid plaque components. For multi-modal MRI images, different modalities can show the degree of lumen stenosis and the plaque shape and its component structure. In the identification and analysis of the biological characteristics of carotid atherosclerosis, medical histopathology and MR imaging technology have a high consistency, which is an effective detection method for the diagnosis and evaluation of carotid plaque. Multimodal MRI have some problems, such as inconsistencies in the image size, scanning orientation, and imaging morphology of the carotid artery. It is necessary to perform unified image registration for images of different modalities based on the spatial organization and anatomical information.

This paper studies the registration problem of multi-modal medical images, designs a network framework for image registration with cross-scale inputs. Our algorithm combines multi-scale features without increasing the number of parameters. Gaussian smoothing loss function is applied to achieve good registration performance. A large number of experiments have proved the effectiveness of the designed algorithm; however, the research work in this article still has limitations. Firstly, for learning methods, the weakly supervised registration framework highly relies on the segmentation accuracy of fixed and moving images to achieve effective performance, so this method may encounter difficulties when encountering imperfect or limited data labels. Secondly, although Siamese network registration structure realizes the registration of different sizes between fixed images and moving images, the network framework is not adaptable to various sizes. If the internal size of the floating image or the fixed image is different, it still not to achieve registration, this method can only be limited to cross-scale registration under the uniform size of the image.

Due to the lack of reliable image similarity measures or automatic landmark extraction methods, automatic multi-modal medical image registration has traditionally been challenging. In this work, we use training image pairs with only sparse annotations and perform registration with cross-scale inputs. This allows the proposed method to be widely used in clinical applications. The experimental results on brain MRI registration prove its generalization. Our future work will study how to make use of sparse training and validation labels to predict dense correspondences in more medical imaging modalities.

## Conclusion

In this work, we have proposed a cross-scale multi-modal image registration method based on Siamese network. The network uses different sizes of registration image input sub-networks to extract various image features, and a simple padding module is designed to make the network can be trained on features of different sizes. In addition, in order to improve the registration performance, a multi-scale loss function under Gaussian smoothing is applied for training optimization. The experimental results show that our method can still guarantee the registration performance in the case of different-size registration after applying the padding module, and the proposed loss function can greatly improve the registration accuracy. We believe that the proposed technique will contribute the diagnosis and quantification of carotid atherosclerosis since it is easy to find calcium, lipid, fibrous cap if multi-modal carotid artery images are well-aligned.

## Data Availability Statement

The original contributions presented in the study are included in the article/supplementary material, further inquiries can be directed to the corresponding author.

## Author Contributions

XH, LM, and MS performed experiments. All authors contribute to design, analysis and writing of this work.

## Funding

This work was supported in part by the Natural Science Foundation of Zhejiang Province under grants LQ20H160052, LY18F030019, LY18F020030, and LY19F030015, in part by the National Natural Science Foundation of China under grants 11302195, U20A20196, U1908210, 61401397, and 61976191.

## Conflict of Interest

The authors declare that the research was conducted in the absence of any commercial or financial relationships that could be construed as a potential conflict of interest.

## Publisher's Note

All claims expressed in this article are solely those of the authors and do not necessarily represent those of their affiliated organizations, or those of the publisher, the editors and the reviewers. Any product that may be evaluated in this article, or claim that may be made by its manufacturer, is not guaranteed or endorsed by the publisher.
